# Evaluation of therapeutic potentials of plant extracts against poultry bacteria threatening public health

**DOI:** 10.1186/s12906-016-1399-z

**Published:** 2016-10-26

**Authors:** Moses Abiala, John Olayiwola, Oluwatoyin Babatunde, Olapeju Aiyelaagbe, Sunday Akinyemi

**Affiliations:** 1 Department of Biological Sciences, Ajayi Crowther University, Oyo, Nigeria; 2 Department of Biological Sciences, Mountain Top University, Prayer City, Nigeria; 3 Department of Chemical Sciences, Ajayi Crowther University, Oyo, Nigeria; 4 Department of Chemistry, University of Ibadan, Ibadan, Nigeria; 5 Fruits and Biotechnology Unit, National Horticultural Research Institute, Ibadan, Nigeria

**Keywords:** Poultry, Antimicrobial, Multidrug resistant bacteria, Extracts, Public health

## Abstract

**Background:**

Plant extracts were evaluated on poultry bacteria known to be threatening public health. This is to develop better bio-therapeutic agents from plant origin.

**Methods:**

Bacteria were isolated from water, feed, crop, gizzard and faeces of layer chicken. Isolates of interest (*Escherichia coli*, *Salmonella enteritidis*, *Pseudomonas aeruginosa* and *Klebsiella oxytoca*) were subjected to antibiotic susceptibility test. Resistant strains were further evaluated against different plant extracts in comparison to Meropenem (control) using agar diffusion method.

**Results:**

*E. coli* had the highest occurrence (53 %), followed by *P. aeruginosa* (25 %) and then *S. enteritidis* (13 %) while the least was *K. oxytoca* (9 %). Virtually all the isolates exhibited multi-antibiotic resistance (MAR) with gross resistance to Amoxicillin, Erythromycin and Cefuroxine. *P. aeruginosa* (75 %), *S. enteritidis* (75 %) and *E. coli* (63 %), had the highest MAR. Out of the 11 (100 %) plant extracts evaluated, 7 (64 %) were outstanding and showed varied levels of antibacterial activity. Specifically, methanol extract of *Mangifera indica* Julie cultivar leaf (MJLM) had the highest antibacterial activity, followed by *Euadenia trifoliata* stem bark (TB03) and *Euadenia eminens* leaf (TB05). *P. aeruginosa* was highly susceptible (81.81 %) to the extracts, followed by *S. enteritidis* (63.64 %) and then *E. coli* (27.27 %).

**Conclusions:**

MJLM and other extracts have proven to be promising extracts in which to search for bioactive components that can be developed into therapeutic drugs. This may help in the management of antibiotic resistant bacterial isolates from poultry chicken threatening public health.

## Background

Poultry is one of the world’s fastest growing sources of meat, and its share in the world meat production has increased from 15 % to 30 % [1]. The modern production unit can produce market ready broiler chicken in less than six weeks. This development arose from genetic selection, improved feeding and health management practices involving use of antibiotics as therapeutic agents [2, 3]. Antibiotics have improved poultry performance effectively and economically [3, 4] but an increase in numbers of antibiotic-resistant bacterial strains [5] such as *E. coli* [6–8], *Salmonella* spp. [9] and *Pseudomonas* spp. [10] continue to occur [4, 11].

The high incidence and rising frequency of antibiotic resistance among the bacteria populating poultry chicken presents a public health hazard [5]. These antibiotic resistant bacteria can be transmitted from poultry to humans through the food chain with serious consequences on public health [5, 12]. This, therefore, necessitates a need for better antimicrobial drugs. Plants have been documented as one of the sources that possesses antimicrobial traits which are chiefly synthesized during secondary metabolism [13–15]. Plant based antimicrobial compounds have great therapeutic potentials as they can serve the purpose without any side effects associated with synthetic drugs [16]. The inherent utility and practical applications of plant extracts such as garlic, cinnamon, tulsi, ginger, turmeric, lemon, neem, yucca, thyme and rosemary have been explored for improving poultry health as well as production with fruitful results [17, 18]. Though, it has been reported that some plant based chemotherapeutic agents may be ineffective on emerging resistant bacterial strains [19, 20], therefore, further work still needs to be done to search for more effective plant based chemotherapeutic agents especially for poultry chicken management.

Generally, plants contain bioactive components [21, 22], that are less toxic and environmentally friendly [23]. Their antibacterial activity have been documented against methicillin-resistant *Staphylococcus aureus* and a variety of other bacteria [24, 25] such as *Escherichia coli*, *Klebsiella pneumonia* [26], *Pseudomonas aeruginosa*, *Proteus mirabilis* [27], *Brevibacterium ammoniagenes*, *Streptococcus mutans* and *Propionibacterium acnes* [28]. Despite the fact that global interest has been shifted to plant based antimicrobials [29], many plants still remain largely untapped against antibiotic resistant bacterial isolates. This study therefore investigated not only susceptibility of isolated bacteria to different antibiotics but also antibacterial activity (in vitro) of plant extracts on selected antibiotic resistant bacterial isolates. These isolates were obtained from water, feed, crop, gizzard, and faeces of poultry layer chicken. The isolates were characterized and assayed as multi-antibiotic resistant bacteria and then subjected to different plant extracts. Apart from *Mangifera indica* (Mango), other plant extracts used have not previously been evaluated on the antibiotic resistant bacterial isolates from poultry chicken.

## Methods

### Collection of samples, bacterial isolation and identification

Poultry practicing rural community, Ilora in Oyo State, Southwestern Nigeria was used as our collection site. The following samples, namely; water, feed, crop and gizzard contents and faecal materials were randomly collected in 3 replicates per sample from at least 5 different poultry farms that were about 100 km apart. Water samples were aseptically collected from nipples, reservoirs and storage tanks of layer chickens. Feed samples were obtained from layer chicken feed while the crop and gizzard were obtained from already sacrificed layer chickens. Fresh faecal materials were also collected from droppings of layer chickens kept in battery cage system. After collection, samples were aseptically transported to laboratory for specific bacterial contents. Apart from water samples, stomacher (Seward Stomacher*80 Lab System, England) at 60 s normal speed was used to aid maceration of solid samples in stomacher bag (1 g in 9 ml of sterile water) for successful serial dilution–pour plate isolation [30] of bacteria. Inoculated nutrient agar (LabM Limited, Lancashire, United Kingdom) and MacConkey agar (Medical Market Limited, United Kingdom) plates were incubated at 37 ± 2 °C for 24–48 h [31]. Distinct colonies were randomly selected and were streaked onto new plates until pure cultures were obtained. The bacterial isolates were identified based on morphological and biochemical tests. Gram and endospore staining, catalase, oxidase, indole and citrate test were carried out following standard protocols [32]. Utilisation of lactose, sucrose, glucose and gas production was confirmed using triple sugar iron (TSI; LabM Limited, Lancashire, United Kingdom) agar slant [33]. Motility test was also carried out by stabbing sulphide indole motility (SIM; Rapid Lab. Colchester Essex, United Kingdom) agar vertically with each bacterium isolate and appearance of turbidity confirmed motility after incubation for 24–48 h [33]. The overall morphological and biochemical characteristics were subjected to identification using Bergey’s Manual of Determinative Bacteriology [34].

### Antibiotic resistance profile of test strains

Prior to antibiotic sensitivity test, McFarland standard corresponding to 0.5 was prepared [35, 36]. Turbidity was confirmed to have optical density (OD) of 0.08–0.10 at 625 nm using photo-electric colorimeter (Callen Komp, England). The antibiotics susceptibility of the isolates was performed using Gram specific antibiotics (Rapid Labs, United Kingdom) which include: Ceftazidime (30 μg), Ciprofloxacin (5 μg), Ofloxacin (5 μg), Amoxicillin (30 μg), Ampicillin (10 μg), Nitrofurantoin (300 μg), Gentamicin (10 μg), Cefuroxime (30 μg), Cloxacillin (5 μg), Erythromycin (10 μg) and Ceftriaxone (30 μg). Using a sterile inoculating loop, the distinct colony of the isolates was emulsified in 3–4 ml of sterile physiological saline and turbidity of the bacterial suspension was matched to the turbidity of the standard. The bacterial suspension was swabbed with sterile cotton swab evenly on Mueller Hinton (MH; Cypress Diagnostic, Belgium) agar in Petri - dishes by rotating plate at approximately 60°. The multi - antibiotic discs (Rapid Labs, United Kingdom) was placed aseptically onto inoculated three replicated plates with respect to each isolate using sterile forceps and incubated aerobically at 37 ± 2 °C for 24–48 h [32, 37]. Diameter of the zones of inhibition were measured with a ruler and recorded in millimeter (mm). The interpretation of the results was done using interpretative chart according to CLSI [38]. Based on the interpretation, the bacterial isolates were classified either as susceptible or resistant.

### Plant preparation and extraction

The leaves and stem bark of three cultivars (Julie, Edward, and Ogbomosho) of *Mangifera indica* were collected from cultivated Mango orchards at the National Horticultural Research Institute (NIHORT), Ibadan in Nigeria and were authenticated by the scientist in charge. It was then identified at the herbarium unit of the Department of Botany, University of Ibadan, Nigeria. The herbarium number were as follows: Julie cultivar - UIH 22514J, Edward cultivar - UIH 22514E, and Ogbomoso cultivar - UIH 22514O. The stem-bark of *Erythrophleum suaveolens* (ID: IFH-17542) as well as the leaves and stem-bark of *Euadenia eminens* (ID: IFH-16540) and *Euadenia trifoliata* (ID: IFH-16539) were collected at a farmland in Lalupon village, Oyo State, Nigeria and were confirmed at herbarium unit, Department of Botany, Obafemi Awolowo University, Ife, Nigeria. The milled, air dried plant samples were extracted with methanol. The extracts obtained were filtered and concentrated under vacuum to give the respective crude extracts. The following codes were given to the extracts: *Erythrophleum suaveolens* leaves (TB01), *Euadenia trifoliata* leaves (TB02), *Euadenia trifoliata* stem-bark (TB03), *Euadenia eminens* stem-bark (TB04), *Euadenia eminens* leaves (TB05), methanol extract of Mango Juliet cultivar leaves (MJLM), methanol extract of Mango Juliet cultivar stem-bark (MJSBM), methanol extract of Mango Ogbomoso cultivar stem-bark (MOSBM), methanol extract of Mango Edward cultivar stem-bark (MESBM) and methanol extract of Mango Edward cultivar leaves (MELM).

### Antimicrobial activity of plant extracts

In preparation for the antimicrobial assay, 50 ml of DMSO (Dimethyl sulfoxide, Sigma-Aldrich) was added to 1 g of each extract in sterile vial bottles to give a stock solution of 1 g/50 ml (w/v) concentration and kept at 4 °C until use. Antibiotic disc diffusion assay [37] with slight modifications [39, 40] was used. Briefly, 24 h old bacterial isolates were standardised against 0.5 McFarland standard [35, 36]. The sterile swab stick was used to pick inoculum from already standardised broth culture in peptone water, and was evenly spread on MH (Cypress Diagnostic, Belgium) agar plates. Sterilised 8 mm cork borer was used to make a hole in the three replicated agar plates and filled with 0.1 ml of each extract (1 g/50 ml equivalent to 0.02 g/ml) while 0.1 ml of 0.02 g/ml of Meropenem (Ranbaxy Laboratories limited, India) was used as the control. The plates were incubated at 37 ± 2 °C for 24–48 h. The diameter of the zone of inhibition around each hole was measured in millimeters (mm) and the mean value was calculated.

### Quantitative evaluation of antimicrobial activity

Percentage activity: This demonstrates the total antimicrobial potency of a particular extract. It shows number of bacteria found susceptible to one particular extract [41].

$$ \mathrm{Activity}\left(\%\right)=\frac{100 \times \mathrm{no}.\ \mathrm{o}\mathrm{f}\ \mathrm{susceptible}\ \mathrm{strains}\ \mathrm{t}\mathrm{o}\ \mathrm{a}\ \mathrm{specific}\ \mathrm{extract}}{\mathrm{Total}\ \mathrm{no}.\ \mathrm{o}\mathrm{f}\ \mathrm{t}\mathrm{ested}\ \mathrm{bacterial}\ \mathrm{strains}} $$

Bacterial susceptibility index (%) (BSI): This is used to compare the relative susceptibility between bacterial isolates. The BSI values range from 0% (resistant to all samples) to 100% (susceptible to all samples) [41].

$$ \mathrm{B}\mathrm{S}\mathrm{I} = \frac{100 \times \mathrm{no}.\ \mathrm{of}\ \mathrm{extracts}\ \mathrm{effective}\ \mathrm{against}\ \mathrm{each}\ \mathrm{bacterial}\ \mathrm{strain}}{\mathrm{Total}\ \mathrm{number}\ \mathrm{of}\ \mathrm{extracts}} $$

### Statistical analyses

Treatments were compared using SAS software, version 9.1 (SAS Institute, Cary, NC, USA) [42]. From in vitro antibiotic susceptibility assay, dependent variables were subjected to analysis of variance (ANOVA), followed by *post hoc* pairwise comparisons using the Student-Newman-Keuls multiple-range test. Three out of the four isolates with high MAR were subjected to eleven extracts in an in vitro experiment. Values obtained from the effectiveness of plant extracts on the isolates were also compared by ANOVA and Student-Newman-Keuls test.

## Results

### Characteristics and occurrence of bacterial isolates

A total of thirty-two Gram negative bacteria were isolated from different poultry chicken sources. The isolates exhibited morphological characteristics that ranges from green, light yellow, deep yellow, rough surface and opaque to circular (Table 1). Bacterial isolates were identified based on morphological characteristics and biochemical test with key interest in *P. aeruginosa*, *S. enteritidis*, *E. coli* and *K. oxytoca* (Table 2). *P. aeruginosa* and *K. oxytoca* were predominantly isolated from water used in poultry chicken management. *E. coli* was isolated from crop, gizzard and poultry chicken faeces while only *S. enteritidis* originated from chicken faeces. Irrespective of the sources, *E. coli* dominated, followed by *P. aeruginosa* while *S. enteritidis* and *K. oxytoca* were the least.

**Table 1:** Characteristics revealing the identity of bacterial isolates from chicken sources

Characters	*P. aeruginosa*	*S. enteritidis*	*K. oxytoca*	*E. coli*
Number of Isolates	08	04	03	17
Pigmentation	Green colonies with rough and spread surface	Creamy, dull, transparent, entire, circular and raised	Mucoid colonies appears in light yellow	Opaque colonies with deeper yellow colour
Gram reaction, Cell shape	-, rod	-, rod	-, rod	-, rod
Catalase	+	+	+	+
Oxidase	+		-	+
Citrate	-		+	+
Motility	+	+	+	+
Hydrogen sulphide production	-	-	-	-
Indole Production	-	-	+	-
Gas production	-	+	+	+
Carbohydrate Utilization:				
i. Glucose	-	+	+	+
ii. Lactose	-	-	+	+
iii. Sucrose	-	+	+	+

**Table 2:** Occurrence of bacteria based on different poultry sources

Isolates	Water	Feed	Crop	Gizzard	Faeces
*P. aeruginosa*	+	+	-	-	-
*S. enteritidis*	-	-	-	-	+
*K. oxytoca*	+	-	-	-	-
*E. coli*	-	-	+	+	+

+ Present, - Absent

### Antimicrobial sensitivity assay

The phenotypic antibiotic resistance profile of the Gram negative bacteria isolated from poultry chicken sources was studied using 50 % as the breaking point for the percentage effectiveness. There was 87–100 % resistance to Cefuroxine across the bacteria except *S. enteritidis* that showed no resistance, however, 75–100 % resistance to Amoxicillin. All the isolates showed gross resistance to Amoxicillin, Erythromycin and Cefuroxine. Surprisingly, *K. oxytoca* showed outstanding sensitivity to Ceftazidine and Cloxacillin in comparison to other isolates that were resistant to them. *P. aeruginosa* and *S. enteritidis* displayed resistance to Gentamycin and they both similarly had the highest MAR (75 %), followed by *E. coli* (63 %) (Table 3).

**Table 3:** Phenotypic antibiotic profiles of the bacteria isolated from different poultry sources

Antibiotics	Code	Potency	*P. aeruginosa* (%)	*S. enteritidis* (%)	*K. oxytoca* (%)	*E. coli* (%)
Ofloxacin	OFL	5 μg	S	S	R	S
Amoxicillin	AUG	30 μg	R	R	R	R
Erythromycin	ERY	10 μg	R	R	R	R
Ceftazidine	CAZ	30 μg	R	R	S	R
Cefuroxine	CRX	30 μg	R	R	R	R
Gentamycin	GEN	10 μg	R	R	S	S
Cloxacillin	CXC	5 μg	R	R	S	R
Ceftriaxone	CTR	5 μg	S	S	S	S
MAR (%)			75	75	50	63

### Antibacterial activity of the plant extracts

**Fig. 1:**
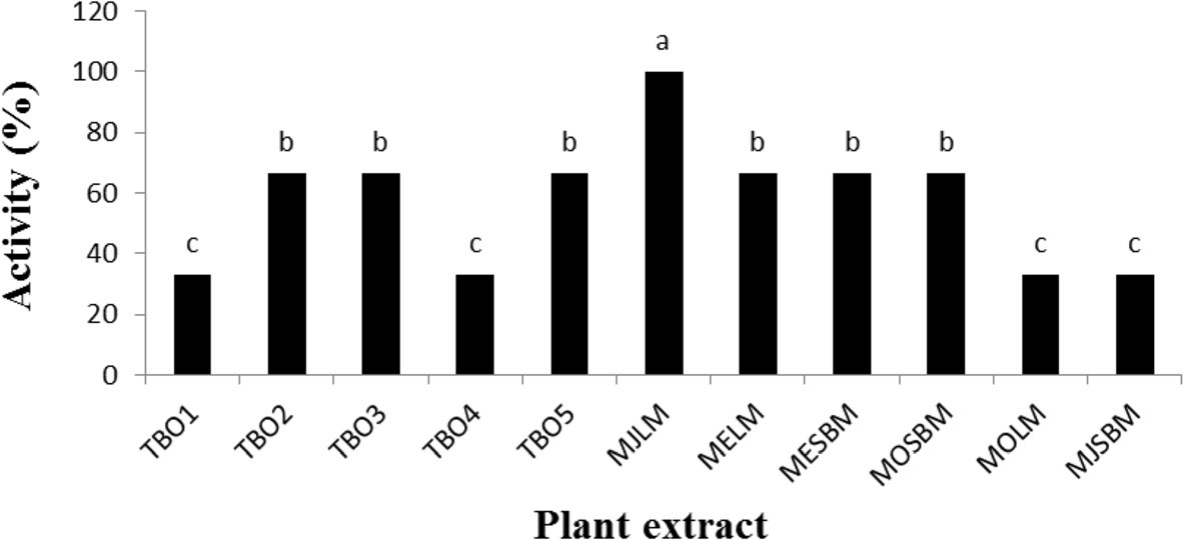
Activity of plant extracts against test isolates (%). Mean values for three replicates are shown. Different letters above the bars indicate significant differences according to the Student Newman-Keuls multiple-range test (0.05)

**Fig. 2:**
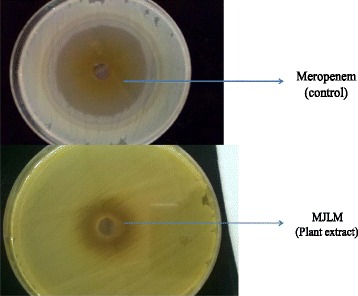
In vitro effect of methanol extract of MJLM (*Mangifera indica*) on *P. aeruginosa*

**Fig. 3:**
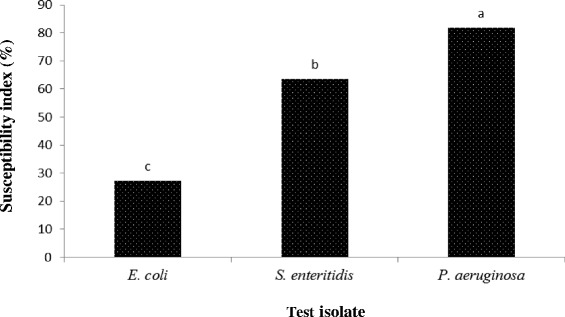
Bacteria susceptibility index (%). Mean values for three replicates are shown. Different letters above the bars indicate significant differences according to the Student Newman-Keuls multiple-range test (0.05)

Bacterial isolates (*P. aeruginosa*, *S. enteriditis* and *E. coli*) that showed high MAR were selected and subjected to different plant extracts. Out of the 11 extracts evaluated, 7 (64 %) were effective and vary in their antibacterial activities against MAR isolates (Table 3). Based on percentage activity, MJLM had the highest antibacterial activity (100 %), followed by TB02, TB03, TB05, MELM, MESBM and MOSBM (66.7 %) while the least were TB01, TB04, MOLM and MJSBM (33.33 %) (Fig. 1). MJLM exhibited broad spectrum inhibitory activity and also compared favourably with Meropenem (control) against the selected MARS bacterial isolates (Table 3; Fig. 2). Specifically, TB01, TB02, TB03, TB05, MJLM, MELM, MESBM, MOSBM and MJSBM were effective on *Pseudomonas* spp. TBO3, MESBM, MOSBM and MOLM demonstrated partial inhibitory activity on *S. enteritidis* in comparison to *E. coli* that exhibited resistance to virtually more than 60 % of the plant extracts (Table 4; Fig. 3).

**Table 4:** Evaluation of plant extracts using agar diffusion method

Plant extracts	Zone of inhibition (mm)		
	*E. coli*	*S. enteritidis*	*P. aeruginosa*
Meropenem (0.02 mg/ml) (Positive control)	22.80 (0.43) a	28.80 (0.21) a	30.60 (0.01) a
DMSO (Negative control)	0.00 (0.00) e	1.00 (0.01) i	1.00 (0.01) h
Distilled Water (Neutral control) (0.02 mg/ml)	0.00 (0.00) e	0.00 (0.00) j	0.00 (0.00) i
TBO1	0.00 (0.00) e	0.00 (0.00) j	6.80 (0.02) c
TBO2	0.00 (0.00) e	3.78 (0.21) g	6.80 (0.01) c
TBO3	3.50 (0.01) c	7.30 (0.12) b	0.00 (0.00) i
TBO4	0.00 (0.00) e	0.00 (0.00) j	4.00 (0.04) g
TBO5	3.35 (0.03) d	0.00 (0.00) j	5.00 (0.04) f
MJLM	7.25 (0.10) b	5.32 (0.21) c	9.80 (0.42) b
MELM	0.00 (0.00) e	2.30 (0.00) h	6.30 (0.07) d
MESBM	0.00 (0.00) e	5.00 (0.02) d	6.50 (0.01) cd
MOSBM	0.00 (0.00) e	4.50 (0.01) e	6.00 (0.02) e
MOLM	0.00 (0.00) e	4.00 (0.01) f	0.00 (0.00) i
MJSBM	0.00 (0.00) e	0.00 (0.00) j	6.50 (0.04) cd

Results are means (standard deviations) for three replicates. Values followed by different letters within a column indicate significant differences according to the Student-NewmanKeuls multiple-range test (0.05)

## Discussion

Interest in poultry bacteria specifically those that threaten public health motivated this study. Based on phenotypic antibiotic susceptibility test, the level of resistance exhibited by the test isolates on selected antibiotics is alarming. This is an indication that indiscriminate use of conventional antimicrobials has led to a steady increase in the antibiotic resistance and the low-income countries which are home to the majority of the world’s population are particularly affected by this phenomenon [14]. With the exception of *K. oxytoca*, the antibacterial activity of plant extracts was centred on *E. coli, S. enteritidis* and *P. aeruginosa* because of their high MAR.

Apart from the occurrence of *E. coli* in the crop, gizzard and faeces of poultry chickens, their resistance to antibiotics is a great concern. This shows that preventive medications are still given to chicken in order to reduce mortality [43]. The application of antibiotics will not only increase the resistance in pathogenic strains but can also lead to resistance in the endogenous flora of humans and animals [44]. Following the consumption of poultry meat specifically chicken, MAR bacterial strains may spread to the human population, which can lead to the transfer of genes coding for resistance [45]. Our observation shows that the resistance rate of *E. coli* to Amoxicillin, Erythromycin, Cefuroxine, Ceftazidine and Cloxacillin presents a serious cause for concern considering the fact that the uninformed farmers may continue to use increasing level of ineffective antibiotics in the management of infection in farms with possible residues in poultry meat, eggs and other products meant for human consumption [46].

One of the ways to handle antibiotic resistant *E. coli* is to develop new and novel antimicrobial agents specifically from plant origin. From this study, the methanol extract of *Mangifera indica* Julie cultivar leaves demonstrated antibacterial effectiveness on antibiotic resistant *E. coli* [47]. This is an indication that the leaves of *Mangifera indica* contain inhibitory substances active against *E. coli* [48] in comparison to *Euadenia trifoliata* that was ineffective. This observation is similar to that of *Euadenia eminens* root extract evaluated against *E. coli* by Dickson et al. [49] but contrary to that of Amole et al. [50]. The resistance of *E. coli* to the extracts [51] is similar to what was obtained for Amoxicillin, Erythromycin, Cefuroxine, Ceftazidine and Cloxacillin. Although, in this case, the crude extract used may not be active enough as potency of each phytoactive components may have been affected by each other. This suggests that further work still needs to be done to evaluate the specific phytoactive compound present in all the tested plant extracts so as to ascertain their efficacy on this antibiotic resistant *E. coli.*

*S. enteritidis* occurrences in faeces of poultry chicken may be as a result of improperly disposed poultry wastes [52]. With reference to Nigeria, wastes from commercial poultry farms are not properly disposed and most rural farmers use these wastes as manure, which are often kept at the backyards before moving them to the farm. These poultry wastes may serve as source of enteric organisms that habour novel resistance factors for birds, including chicken that feed on such wastes. The isolated *S. enteritidis* was generally resistant to Gentamycin, Amoxicillin, Cloxacillin, Erythromycin, Cefuroxime and Ceftazidine. The observed high resistance against these antibiotics probably reflects the high usage of the drugs in the study sites. This could be because these antibiotics are readily available and farmers see them as first point of contact broad-spectrum to treat their chickens [53, 54]. As a result of this, these drugs may have become seriously compromised and probably are currently ineffective.

The high rates of antibiotic resistance in *S. enteritidis* may be more difficult to treat with synthetic antibiotics alone, but rather needs the touch of nature such as bioactive components in plants. Evaluated plant extracts inhibited the growth of *S. enteritidis* in vitro. Extracts from *Euadenia trifoliata* leaves and *Euadenia trifoliata* stem-bark demonstrated antibacterial activity against *S. enteritidis*. This corroborated the work of Amole et al. [50] using *petroleum ether, ethyl acetate and methanol fractions of Euadenia trifoliata leaves* against *S. typhi.* Osundiya et al. [55] also reported similar observation using *root* extracts of *Burkea africana* and *Combretum adenogonium* against *S. typhi*. The antibacterial activity of methanol extract of *Mangifera indica* - Julie cultivar leaves on *S. enteritidis* is in agreement with the work of De and Pal [48] who also used *aqueous young leaves extract of Mangifera indica* against *S. typhi*. This suggests that *Euadenia trifoliate* and *Mangifera indica* possesses antibacterial compounds which are active against *Salmonella* species.

The occurrence of *P. aeruginosa* in feed [56] and water [10, 57] is not new however, our work re-established it. *P. aeruginosa* exhibited resistance to Erythromycin, Amoxicillin, Cloxacillin, Cefuroxime, Ceftazidine and Gentamycin which corroborated with the work of Kibret and Abera [58]. *Pseudomonas* species are naturally resistant to many antibiotics due to the permeability barriers afforded by its outer membrane composed of lipopolysaccharide [27]. Resistance of *P. aeruginosa* to Gentamycin, is unique because it has been documented that *P. aeruginosa* used to be resistant to certain antibiotics [59] but not Gentamycin, however this may be peculiar to our study site. This suggests that poultry chicken farmers (especially the untrained) in the study site may have indirectly abused Gentamycin as a broad spectrum antibiotic [53, 54] to treat poultry chicken diseases.

*P. aeruginosa* was resistant to more than 70 % of the antibiotics, but sensitive to more than 80 % of the extracts evaluated. This shows that one of the methods to reduce the resistance of *P. aeruginosa* to synthetic antibiotics is by using antibiotic resistant inhibitors from plant origin [60, 61]. The effectiveness of the plant extracts is in agreement with Abid et al. [62] who also emphasized that the most sensitive bacterium to tested plants is *Pseudomonas*. In this study, activity of *Euadenia trifoliata* [50], *Euadenia eminens* [49] and *Mangifera indica* was evident against *P. aeruginosa* [62]. Interestingly, methanol extracts of *Mangifera indica* (MJLM) showed more activity against *P. aeruginosa* [47] in comparison to other extracts. This suggests that *Mangifera indica* possesses potent and specific inhibitory substance (s) against *P. aeruginosa*.

## Conclusions

Isolated bacteria were resistant to more than two antibiotics. Apart from gross resistance to Amoxicillin, Erythromycin and Cefuroxine, susceptibility to Ceftriaxone was observed across the bacteria investigated. The in vitro study revealed that 70 % of extracts exhibited antibacterial activity against test isolates. MJLM and other extracts have proven to be promising extracts in which to search for bioactive compounds that can be developed into therapeutic drugs. These, therefore, substantiate their therapeutic use in the control of antibiotic resistant *P. aeruginosa*, *S. enteriditis* and *E. coli* threatening public health through poultry chicken.
